# Profiling of chicken adipose tissue gene expression by genome array

**DOI:** 10.1186/1471-2164-8-193

**Published:** 2007-06-27

**Authors:** Hong-Bao Wang, Hui Li, Qi-Gui Wang, Xin-Yu Zhang, Shou-Zhi Wang, Yu-Xiang Wang, Xiu-Ping Wang

**Affiliations:** 1 College of Animal Science and Technology, Northeast Agricultural University, Harbin, 150030, P.R. China; 2 Ministry of Education Key Laboratory of Bioinformatics, School of Biomedicine, Tsinghua University, Beijing, 100084, P.R. China; 3 Shanghai Biochip Co., Ltd., Shanghai, 201203, P.R. China

## Abstract

**Background:**

Excessive accumulation of lipids in the adipose tissue is a major problem in the present-day broiler industry. However, few studies have analyzed the expression of adipose tissue genes that are involved in pathways and mechanisms leading to adiposity in chickens. Gene expression profiling of chicken adipose tissue could provide key information about the ontogenesis of fatness and clarify the molecular mechanisms underlying obesity. In this study, Chicken Genome Arrays were used to construct an adipose tissue gene expression profile of 7-week-old broilers, and to screen adipose tissue genes that are differentially expressed in lean and fat lines divergently selected over eight generations for high and low abdominal fat weight.

**Results:**

The gene expression profiles detected 13,234–16,858 probe sets in chicken adipose tissue at 7 weeks, and genes involved in lipid metabolism and immunity such as *fatty acid binding protein (FABP), thyroid hormone-responsive protein (Spot14), lipoprotein lipase(LPL), insulin-like growth factor binding protein 7(IGFBP7) *and *major histocompatibility complex (MHC)*, were highly expressed. In contrast, some genes related to lipogenesis, such as *leptin receptor, sterol regulatory element binding proteins1 (SREBP1), apolipoprotein B(ApoB) *and *insulin-like growth factor 2(IGF2)*, were not detected. Moreover, 230 genes that were differentially expressed between the two lines were screened out; these were mainly involved in lipid metabolism, signal transduction, energy metabolism, tumorigenesis and immunity. Subsequently, real-time RT-PCR was performed to validate fifteen differentially expressed genes screened out by the microarray approach and high consistency was observed between the two methods.

**Conclusion:**

Our results establish the groundwork for further studies of the basic genetic control of growth and development of chicken adipose tissue, and will be beneficial in clarifying the molecular mechanism of obesity in chickens.

## Background

The chicken is an important model organism that bridges the evolutionary gap between mammals and other vertebrates [1]. Research on chickens (*Gallus gallus*) has had a significant impact on fundamental biology. The domestic chicken also provides a major protein source from meat and eggs for most human populations throughout the world. Its economic importance has made it the focus of numerous research projects, including a recent effort to sequence the entire chicken genome [2].

During the past 80 years, selective breeding has made spectacular progress in both egg and meat production traits. The modern commercial broiler is the product of intensive selection for rapid growth and enhanced muscle mass over many generations. Associated with these successes there have been a number of undesirable traits, such as ascites and lameness, reduced fertility, and reduced resistance to infectious diseases [3]. Selection for rapid growth has been also accompanied by increased fat deposition in these animals [4,5]. Excessive fat is a major problem for the modern broiler industry, since it not only reduces carcass yield and feed efficiency, but also causes rejection of the meat by consumers [6] and difficulties in processing [7].

With the rapid development of molecular biotechnology, various studies have been performed to investigate the metabolic and genetic mechanisms involved in the regulation of fatness in chickens. Because *de novo *fatty acid synthesis in birds takes place mainly in the liver, most studies have been performed on hepatic tissue. There have been few analyses of the expression of adipose tissue genes involved in pathways and mechanisms leading to adiposity in chickens. In the present study, Chicken Genome Arrays were used to construct the gene expression profiles of 7-week-old broilers, and to screen genes that are differentially expressed in adipose tissue between lean and fat lines divergently selected over eight generations for high and low abdominal fat weight. Our study will be beneficial in clarifying the molecular mechanisms of obesity in chicken, and these data will contribute to related research on other species.

## Results

### Characterization of the two chicken lines

**Figure 1:**
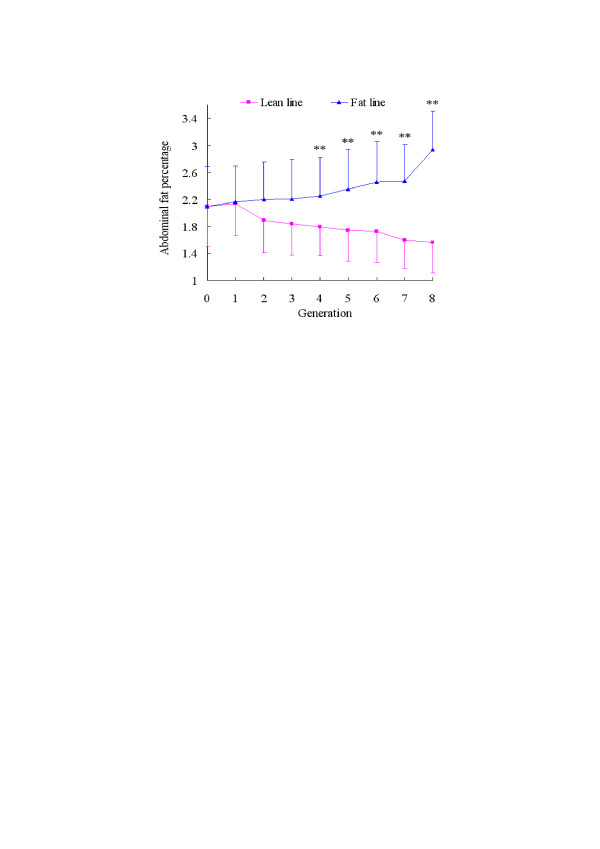
**The separation of AFP between fat and lean lines**. The lean and fat chicken lines derived from a commercial Arbor Acres (AA) grandsire line were created at the Northeast Agricultural University Animal Breeding Center in 1996. They have been selected for 8 generations up to 2004. The selection criteria were the proportion of abdominal fat and levels of very low density lipoproptein (VLDL) in males at 7 weeks of age. Significant differences in AFW and AFP between the two lines were apparent from the 4th generation. Selection was continued for 8 generations, with 15 sires and 4 hens per sire in each line for the G0 to G5 generations and 25 sires and 4 dams per sire in each line for generations G6 to G8. The number of fat line chickens from G1 to G8 was: 82, 88, 75, 81, 80, 78, 179, 165, respectively; and in lean line from G1 to G8: 124, 133, 127, 141, 139, 145, 258, 219, respectively. G represents generation. **p < 0.01, significant difference in AFP between the two lines.

It is clear that the percentages of abdominal fat in the two lines have become very different after selecting for eight generations (Figure 1). In the eighth generation, the AFP is 2.95% in the fat chicken line and 1.55% in the lean line.

**Figure 2:**
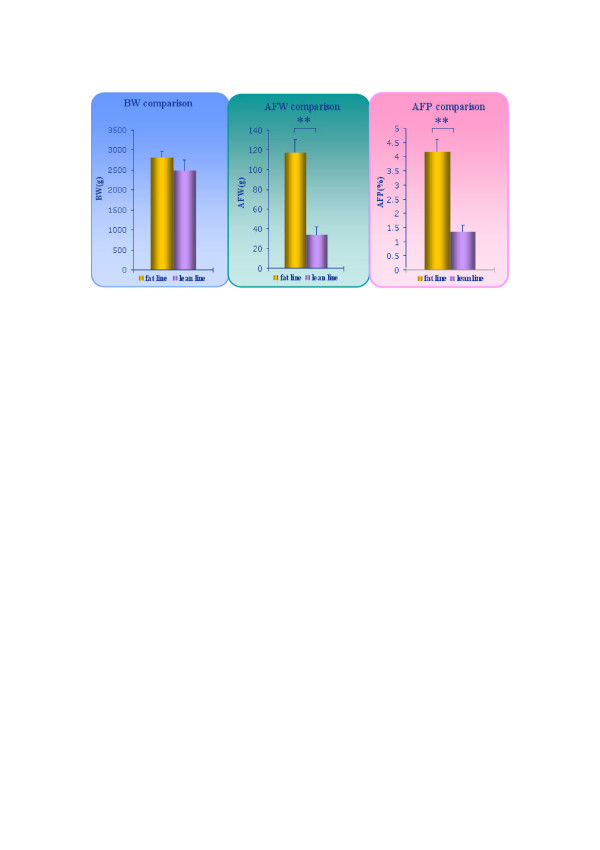
**Comparison of BW, AFW and AFP in chickens used in the study**. The birds were kept in similar environmental conditions and had access to feed and water *ad libitum*, and were fed with a commercial corn-soybean-based diet that met all NRC requirements for 7 weeks. BW was measured after 12 hours fasting, then the birds were slaughtered and the abdominal fat was isolated and weighed. The AFP was calculated as AFP (%) = AFW (g)/BW (g). BW, AFW and AFP data for the ten birds are shown in Table 1. BW is not significantly different between the two lines, but AFW and AFP differ significantly. Data are mean ± SD (n = 5). **p < 0.01, significant difference between fat line and lean line.

The chickens used in this study differed significantly not in body weight but in abdominal fat weight and percentage of abdominal fat (Figure 2) and were chosen on that basis. AFP in the fat line was three times that in lean line (Table 1).

**Table 1:** BW, AFW and AFP of chickens used in the study

	Fat line					Lean line				
	7-3-3	7-3-8	7-3-15	7-3-16	7-3-24	7-1-1	7-1-2	7-1-4	7-1-11	7-1-17
BW(g)	2690	2615	2915	2940	2895	2655	2655	2080	2375	2685
AFW(g)	103.4	116.6	115.3	112.1	138.4	36.91	40.13	28.27	23.46	40.83
AFP (%)	3.84	4.46	3.96	3.81	4.78	1.39	1.51	1.36	0.99	1.52

### Adipose tissue gene expression profile

**Figure 3:**
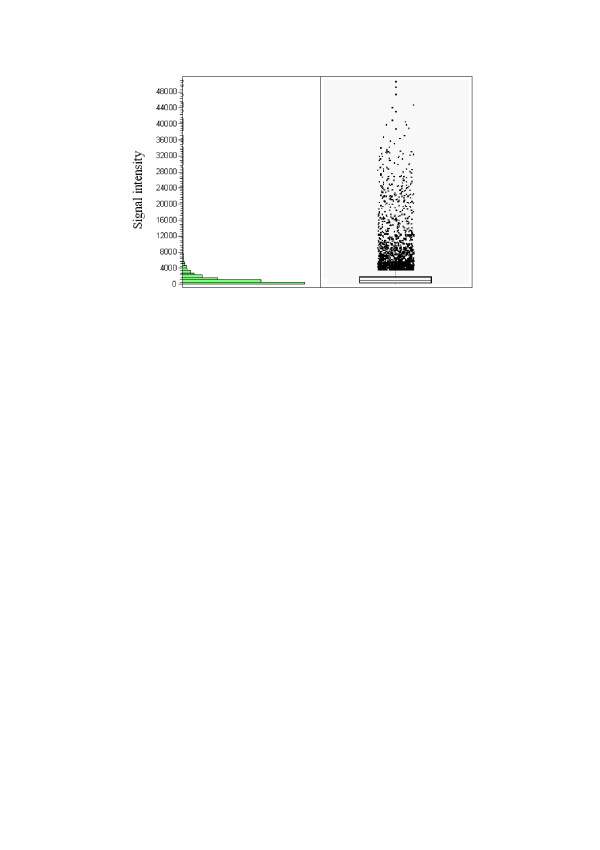
**The distribution of genes expressed in adipose tissue calculated by JMP**. Raw data sets were normalized to total fluorescence, which represents the total amount of cRNA hybridized to a microarray using the software Affymetrix Microarray Suite 4.0. Data sets were excluded if the absolute call (Abs Call) was A (absent) or M (Marginal) according to the detection *p*-value in all arrays. Only expressed transcripts (the Abs Call was P, present) were used in the analysis. The y-axis is the signal intensity after normalization, which represents the relative expression level of genes. The signal intensity of most genes was under 10,000, that most genes are poorly expressed in chicken adipose tissue at 7 weeks.

The pattern of adipose tissue gene expression of chickens at 7 weeks was analyzed by oligonucleotide microarrays. Normalized data were used to analyze the total expressed genes. Depending on the individual bird, 13,234–16,858 probe sets were detected (bird 7-3-16 was excluded from subsequent analysis for the reason given below). Subsequently, the distribution of expression levels of genes in adipose tissue was calculated by JMP4.0 (Figure 3). The genes were ordered according to their mean expression levels; those with expression levels in the highest or lowest 1% were considered highly expressed or the converse. Some of the genes with especially high or low expression levels are named in Tables 2 and 3.

**Table 2:** Some highly expressed genes in chicken adipose tissue at 7 weeks

	The signal intensity analyzed by scanner									
Genes	Fat line					Lean line				
	
	7-3-3	7-3-8	7-3-15	7-3-16	7-3-24	7-1-1	7-1-2	7-1-4	7-1-11	7-1-17
A-FABP	44732	47715	46157	57201	52558	52286	49813	51861	50529	47910
spot 14	33917	30120	28795	35261	36359	19983	25819	24245	31674	31015
LPL	27133	24469	29421	25855	30799	18393	20950	23462	25275	23246
IGFBP-7	20471	22532	21163	27799	24675	24113	25764	29104	26375	28610
BMP7	29042	30313	25289	25124	22610	15932	24165	13953	26518	33345
hsp70	33480	32478	30641	21373	23217	19213	21624	16512	21503	30312
PCHK23	21402	19941	19423	30817	30956	29866	25764	24882	22561	25995
hsp25	32908	19470	23662	21037	25644	19327	7967	22002	19564	27742
glutathione peroxidase 4	22459	21447	24945	23085	24229	18705	23685	27400	27560	24096
stem cell antigen 2	32224	31256	22169	24894	26446	21403	27675	24483	26370	26105
CD74 gene	45284	38191	36116	38875	37099	43381	38466	32027	40065	39493
Vimentin	38624	36822	37965	33403	35772	36159	40251	41728	40184	40972
ubiquitin C	42501	37306	45125	39121	35707	35202	40535	42767	43055	41982
Thymosin beta 4	35340	33804	38768	34644	30105	42836	37625	34939	36087	37120
MCH II antigen B-L Beta	32147	29581	28406	34359	28741	38580	30089	29337	28674	31680
T-cell leukemia, homeobox 3	26240	29169	34325	24093	24060	36533	29476	28294	28493	31986
platelet/endothelial cell adhesion molecule	21515	16459	10731	12262	13868	16684	18476	17751	14270	17975

**Table 3:** Some poorly expressed genes in chicken adipose tissue at 7 weeks

	The signal intensity analyzed by scanner									
Genes	Fat line					Lean line				
	
	7-3-3	7-3-8	7-3-15	7-3-16	7-3-24	7-1-1	7-1-2	7-1-4	7-1-11	7-1-17
IGF-I	5.7	11.3	91	7	5.8	52	27.6	22.7	46.6	91.6
UCP	43.3	87.1	104.2	130	119.4	122.2	818.2	289.2	159.6	150.6
APOVLDLII	140.1	103	75.8	107.3	156.2	298.3	72.5	22.6	143.8	98.4
L-FABP	57.7	70.6	126.9	32.6	128.3	623.8	844.5	88.8	52.4	4865
TGFa	115.1	216.8	79.8	51	94.3	186.6	37.7	49.7	166.1	95.8
TGFβR	82.3	151.7	83.3	153.6	146	279.6	166.3	221.9	140.6	191.4
PEPCK-M	503.8	206.3	326	38.1	117.3	404.2	154.2	150.5	140	198.6
BMP2	236	355.4	262.7	317.2	430.9	209.3	399.6	228.6	337	208.6
acetyl-coenzyme A carboxylase alpha	232.5	181.8	319.5	396.4	172.9	181.7	160.8	207.1	144.9	111.8
type I TGF β receptor	82.3	146	153.6	83.3	151.7	191.4	140.6	221.9	279.6	166.3
cAMP response element- binding protein	96.9	115.5	132.3	75	146.5	171.6	91	82.7	196.4	132.6
hexokinase 2 (HK2)	270.3	458.9	526.4	779.8	287.3	523	471.5	337.2	294.7	398.3
Proinsulin	263	269.5	361	540.1	293	336	311	411.1	422.6	190.2
GDF-9	198.1	197.8	216.7	413	190.7	246.8	151.7	110.6	262.9	147.2
BMP5	216.3	382.1	179.5	241.8	257.8	132.2	282.2	264.5	173	288.6
mitogen-activated protein kinase phosphatase 2	231.5	288.2	247.1	238.6	280.5	239.6	136.8	415.3	300.7	205.1
(MKP-2) riboflavin-binding protein	239.7	17.6	179	75.8	242.5	139.8	424.4	60.8	129.9	291.4

We also believe that genes described as "not expressed" can provide useful information about the function of adipose tissue and lipid metabolism in chicken. Table 4 shows some of these genes, selected using the software Affymetrix Microarray Suite 4.0. Many genes allegedly involved in lipid metabolism and obesity were not expressed in adipose tissue in the 7-week-old chickens.

**Table 4:** Some "not expressed" genes in adipose tissue of 7-week-old chickens

Probe set ID	Genes	mismatching probes pairs/the total probes pairs^a^
Gga.10702.1.A1_at	insulin-like growth factor 2	10/10
Gga.11305.1.S1_s_at	thyroid hormone receptor associated protein 2	10/10
Gga.11817.1.S1_s_at	apolipoprotein B	10/10
Gga.12348.2.S1_a_at	glycosyltransferase	10/10
Gga.13.1.S1_s_at	leptin receptor	10/10
Gga.4123.1.S1_at	lipoprotein (APOVLDLII)	10/10
Gga.1731.1.S1_at	pyruvate carboxylase	10/10
Gga.742.1.S1_at	somatostatin-14	10/10
Gga.1267.1.S1_a_at	growth hormone 1	10/10
Gga.560.1.S1_at	growth differentiation factor 8	10/10
Gga.5646.2.S1_at	diacylglycerol kinase, zeta 104 kDa	10/10
GgaAffx.21833.1.S1_s_at	cholecystokinin receptor	10/10
GgaAffx.21834.1.S1_s_at	cholecystokinin	10/10
GgaAffx.21846.1.S1_s_at	transforming growth factor alpha	10/10
GgaAffx.2229.1.S1_at	polyamine oxidase (exo-N4-amino)	10/10
Gga.579.1.S1_at	neurotrophic tyrosine kinase, receptor, type 1	10/10
Gga.609.1.S1_at	thyroid hormone receptor beta 2	10/10
Gga.151.1.S1_at	sterol regulatory element binding transcription factor 1	10/10
Gga.686.1.S1_at	bone morphogenetic protein 4	10/10
Gga.689.1.S1_at	low density lipoprotein-related protein 1	10/10
Gga.15741.1.S1_at	growth arrest-specific 2	10/10
Gga.16782.1.S1_at	phosphoinositide-3-kinase, catalytic	10/10
Gga.761.1.S1_at	Mel-1c melatonin receptor	10/10
Gga.762.1.S1_at	melatonin receptor 1	10/10
Gga.784.1.S1_at	protein-tyrosine phosphatase CRYPalpha	10/10
Gga.793.1.S1_s_at	acetylcholinesterase	10/10
Gga.811.1.S1_at	growth differentiation factor 2	10/10
Gga.17381.1.S1_at	phospholipase C-like 3	10/10
Gga.857.1.S1_at	epidermal growth factor receptor	10/10
Gga.6214.1.S1_a_at	phospholipase A2, group IB (pancreas)	10/10
Gga.3663.2.S1_at	nucleolin	10/10
Gga.3707.2.A1_at	CCAAT/enhancer binding protein (C/EBP), gamma	10/10
GgaAffx.2229.1.S1_at	polyamine oxidase (exo-N4-amino)	10/10
GgaAffx.3695.1.S1_s_at	myelin transcription factor 1	10/10
GgaAffx.3803.6.S1_at	plexin A1	10/10
Gga.2645.1.S1_at	transforming growth factor, beta receptor II	10/10
Gga.2694.1.S1_at	lymphoid enhancer-binding factor 1	10/10
Gga.271.1.A1_at	bone morphogenetic protein 1	10/10
Gga.2933.1.S1_at	N-acetylglucosaminyltransferase VI	10/10
GgaAffx.20398.1.S1_s_at	phospholipase C-like 3	10/10

a: mismatching probes pairs in the total probes (10 pairs) that represent certain genes.

### Analysis of consistency within the fat and lean lines

**Figure 4:**
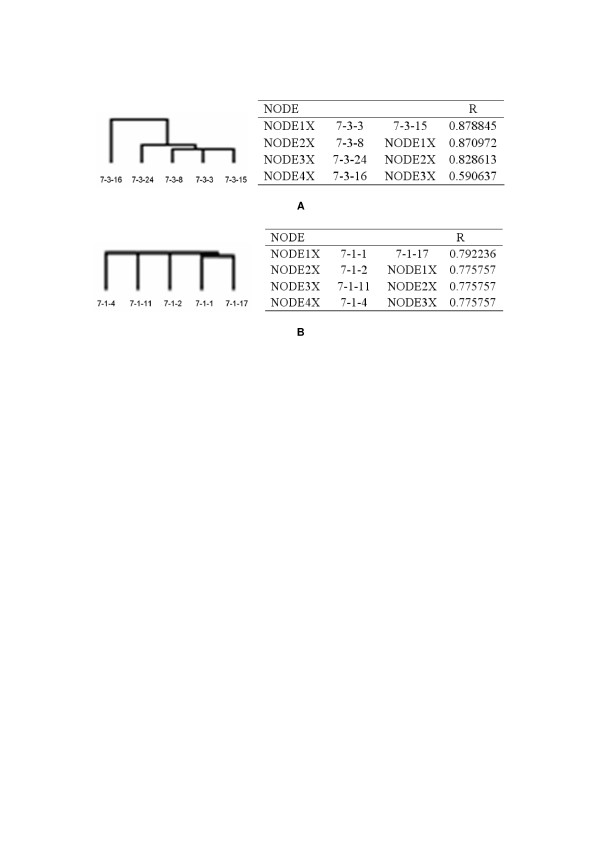
**The hierarchical clustering results of five fat line and five lean line chickens**. A: hierarchical clustering results for five fat line chickens. B: hierarchical clustering result for five lean line chickens. The data used for clustering were normalized data that excluded the "not expressed" genes. R represents the coefficient of correlation among individuals. The fat chicken line shared about 87% consistency, while the lean chicken line shared about 77% consistency. Sample 7-3-16 shared only 59% consistency with the other fat line chickens, so it was excluded from the subsequent screening of differentially expressed genes.

Although there is little difference among individuals in the AFW and AFP within each line, individuals in each line may differ in hereditary molecular characters. By comparing the consistency within each line, the selective effect can be evaluated properly, and chickens that deviate too much from the norm can be excluded from subsequent screening of differentially expressed genes. This makes the analysis of differentially expressed genes more reliable and credible. The cluster analysis results showed that the fat line individuals, except chicken 7-3-16, were more consistent that the lean line ones. Chicken 7-3-16 deviated too much from the other fat line chickens (R<0.6) so it was excluded from the screening of differentially expressed genes. The hierarchical clustering results are shown in Figure 4.

### Identification of differentially expressed genes

**Figure 5:**
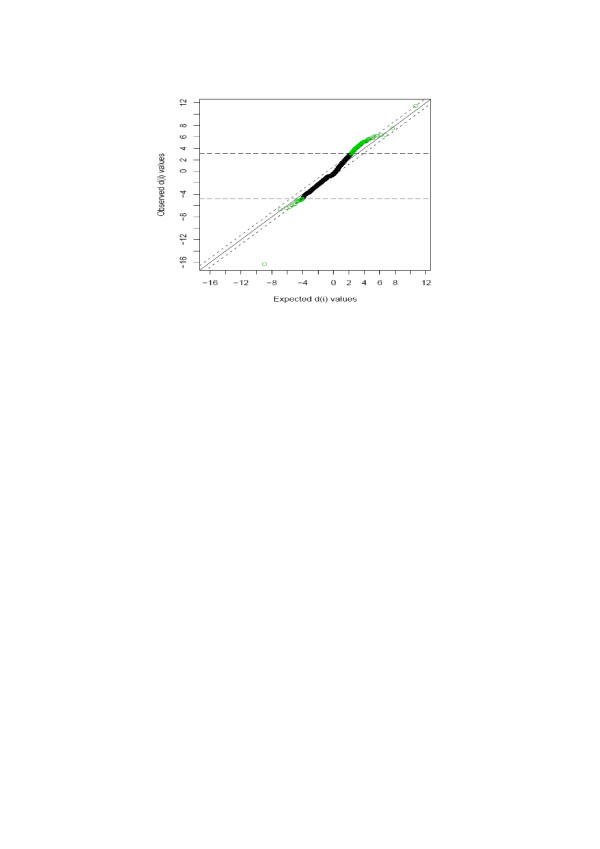
**SAM Plot for Delta = 0.8**. Differentially expressed genes were identified from normalized data using the Significance Analysis of Microarrays (SAM) algorithm implemented in a TIGR MultiExperiment Viewer. A cutoff value delta, depending on an arbitrary false positive rate, was chosen to identify significantly differentially expressed genes. For this analysis, a delta value of 0.8 was used, giving a reasonable cutoff of 3.13 and 4.73 in d-scores.

**Figure 6:**
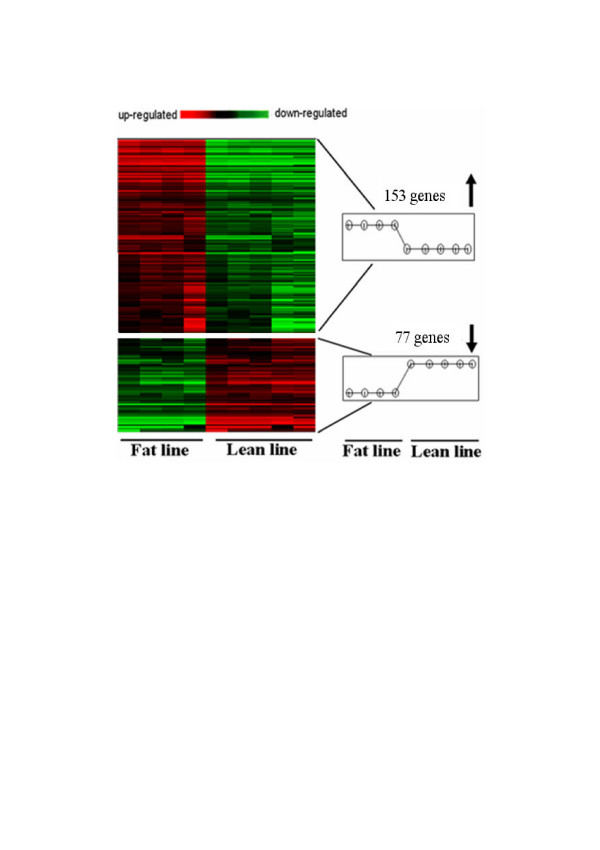
**Cluster image of 230 significant differentially expressed genes**. A total of 230 differentially expressed genes were identified by SAM algorithm: 153 were up-regulated and 77 were down-regulated in fat chickens compared to lean chickens. Colored bars indicate relative expression levels. Genes that are expressed at higher levels are assigned progressively brighter shades of red, while genes expressed at low levels are assigned shades of green.

In order to identify the differentially expressed genes, Significance Analysis of Microarrays (SAM) was performed on normalized data as described by Tusher et al. [8]. To avoid the low-variance problem in t-tests, SAM uses a statistical method similar to the t-test and estimates the false discovery rate by permutation of repeated measurements [8]. Subsequently, a two-class SAM analysis was performed on the log transformed data matrix (see Materials and Methods). A cutoff value, delta, depending on an arbitrary false positive rate, was chosen to identify genes that were significantly differentially expressed. For this analysis, a delta value of 0.8 was used (Figure 5). This led to the identification of a total of 230 differentially expressed genes: 153 were up-regulated and 77 were down-regulated in fat chickens compared to lean chickens (Figure 6). Highly differentially expressed genes were further selected by the fold change (fat/lean). These differentially expressed genes were mainly involved in lipid metabolism, energy metabolism, signal transduction, immunity and tumorigenesis. Table 5 is a summary of the most representative of these genes.

**Table 5:** Differentially expressed genes by microarray analysis

Probe set ID	Fold Change (fat/lean)	T-test P-value	SAM q-value	Chr. Location	GenBank ID	Gene name
**Lipid Metabolism**						
GgaAffx.24836.1.S1_at	2.5	0.001	0.27	chr1: 100587339–100699482	XM_416725	propionyl-Coenzyme A-carboxylase
GgaAffx.22084.1.S1_at	1.8	0	0.27	chr11: 59355–69450	No record	lysophospholipase 3
Gga.150.1.S1_at	4.2	0.003	0.279	chr1: 167849591–167950628	NM_204252	fms-related tyrosine kinase 1 (vascular endothelial growth factor/vascular permeability factor receptor)
GgaAffx.8095.1.S1_at	1.6	0.006	0.324	chr1: 52216531–52245657	XM_416329	N-acetylglucosamine-1-phosphate transferase, alpha and beta subunits
Gga.879.1.S1_at	-6.7	0.001	0.27	chr11: 16001674–16006281	NM_205526	N-acetyltransferase
Gga.19791.1.S1_s_at	1.9	0.003	0.279	chr2: 128797196–128808892	XM_418378	low density lipoprotein-related protein 12
GgaAffx.9942.1.S1_at	2.2	0.001	0.27	chr4: 81245396–81273837	XM_420814	acyl-CoA oxidase
GgaAffx.20938.1.S1_at	1.3	0.007	0.329	chr5: 15830450–15831289	NM_001031187	pyruvate dehydrogenase complex, component X similar to glycerol-3-phosphate dehydrogenase 2;
GgaAffx.23939.1.S1_s_at	7.5	0.005	0.301	chr7: 36699654–36750512	XM_422168	glycerol phosphate dehydrogenase 1, mitochondrial; FAD-linked glycerol-3-phosphate dehydrogenase
Gga.17037.1.S1_s_at	1.6	0.005	0.308	chrZ: 15403025–15434404	NM_001031422	phosphatidylinositol-4-phosphate 5-kinase, type I, beta
GgaAffx.5721.1.S1_at	2.1	0.001	0.27	chr3: 9206064–9214413	NM_001031039	lysocardiolipin acyltransferase
**Energy Metabolism**						
Gga.11825.1.S1_at	3.2	0.003	0.274	chr1: 109208120–109232230	XM_416788	glycerol kinase
Gga.9828.1.S1_a_at	-3.2	0	0.27	chr1: 79394222–79396236	XM_416612	thioredoxin-like 4B
GgaAffx.4389.1.S1_at	-1.6	0.004	0.284	chr10: 17276047–17283495	XM_425083	5-oxoprolyl-peptidase
GgaAffx.2243.2.S1_at	2.3	0.002	0.27	chr26: 3685185–3702282	XM_418055	ATPase, Ca++ transporting, plasma membrane 4
Gga.19534.1.S1_at	8.3	0.002	0.27	chr9: 21559961–21563214	">XM_422814">	beta 1,3-galactosyltransferase, polypeptide 3
**Tumorigenesis**						
Gga.6519.1.S1_s_at	-2.2	0.005	0.303	chr1: 21687403–21784133	XM_416014	suppression of tumorigenicity 7
Gga.15871.1.S1_at	-1.6	0.006	0.323	chr19: 8852117–8853712	CR387378	Tumor necrosis factor, alpha-induced protein 1
Gga.12058.1.S1_at	2.1	0.003	0.279	chr2: 135144222–135145306	XM_418407	similar to cylindromatosis (turban tumor syndrome); cylindromatosis 1, turban tumor syndrome
GgaAffx.22987.1.S1_at	-1.6	0.002	0.27	chr5: 26523325–26529591	XM_421205	thrombospondin 1
Gga.654.1.S2_at	1.8	0.001	0.27	chr6: 4237546–4314610	NM_205190	ret proto-oncogene
**Signal Transduction**						
GgaAffx.3663.1.S1_at	-5.3	0.003	0.278	chr14: 8517712–8520620	XM_414857	similar to CASK interacting protein 1
Gga.12193.1.S1_a_at	9.0	0.007	0.324	chr2: 47562155–47581032	XM_418851	G-substrate
Gga.9510.1.S1_at	1.9	0.007	0.329	chr22:537865–569787	NM_001030886	protein phosphatase 2 (formerly 2A), regulatory subunit B (PR 52), alpha isoform
Gga.1761.2.S1_at	13.8	0.002	0.27	chr3:107742849–107744353	XM_420066	similar to G-protein coupled receptor 116
GgaAffx.4775.1.S1_s_at	4.0	0.002	0.27	chr4:11707299–11760821	XM_420292	similar to receptor tyrosine kinase flk-1/VEGFR-2
Gga.807.1.S1_at	1.9	0.007	0.324	chr4:65686004–65686340	NM_001004368	kinase insert domain receptor (a type III receptor tyrosine kinase) (KDR)
Gga.12481.1.S1_s_at	1.9	0.007	0.327	chr7:27843665–27860405	NM_001012920	protein tyrosine phosphatase-like (proline instead of catalytic arginine), member b
Gga.6653.1.S1_at	-1.7	0.004	0.281	chr11:17426083–17429654	NM_001030576	hepatic nuclear factor 4beta similar to suppressor of cytokine signaling 7; SH2
GgaAffx.8729.1.S1_s_at	-10.0	0	0.27	chrUn:137141542–137146475	XM_423895	domain containing SOCS box protein SOCS7; Nck, Ash and phospholipase C binding protein similar to prostaglandin E receptor 3; Rat kidney
GgaAffx.7207.1.S1_at	5.7	0.002	0.27	chr8:29278163–29285596	XM_426672	prostaglandin EP3 receptor (alternative splicing results in two different receptors EP3a and EP3b);
Gga.10737.1.S1_s_at	1.7	0.005	0.305	chr7:2212065–2223469	XM_421856	similar to Nck-associated protein 1 (NAP 1) (p125Nap1) (Membrane-associated protein HEM- 2)
GgaAffx.8719.1.S1_at	7.2	0.002	0.27	chr4:64869759–64912904	XM_420693	similar to regulator protein p122-RhoGAP – rat
Gga.12408.3.S1_a_at	2.7	0.005	0.305	chr5:41375030–41383659	NM_204357	ataxin 3
Gga.323.1.S1_at	3.6	0.001	0.27	chrUn:147862083–147882739	NM_204801	tetraspanin 18
**Immunity**						
Gga.13806.1.S1_at	-4.8	0.004	0.293	chr7:7186627–7189336	NM_204240	adenosine deaminase, RNA-specific, B1 (RED1 homolog rat)
Gga.18504.1.S1_at	6.9	0.003	0.278	chr5:5440836–5460278	CR390783	parvin, alpha
Gga.698.1.S1_at	1.6	0.004	0.293	chr6:14652488–14704778	NM_205441	vinculin
Gga.11155.1.S1_s_at	1.8	0.001	0.27	chr4:15341234–15354773	XM_420331	stromal antigen 2
**Others**						
Gga.11258.1.S1_at	1.7	0.001	0.27	chr1:170378838–170513289	XM_417140	EF-hand domain family, member A1
Gga.15085.2.S1_s_at	6.6	0.001	0.27	chr1:172635432–172645292	XM_417167	CWF19-like 2, cell cycle control (S. pombe)
GgaAffx.5969.1.S1_at	6.2	0.002	0.27	chr1:24071401–24234367	XM_416024	similar to dedicator of cytokinesis 4
GgaAffx.12338.1.S1_s_at	3.9	0.001	0.27	chr15:7114454–7122253	NM_001030665	tuftelin interacting protein 11
GgaAffx.25724.1.S1_s_at	7.4	0.002	0.27	chr18:5638526–5691321	XM_415648	similar to Stxbp4 protein
GgaAffx.6849.1.S1_at	4.0	0.004	0.285	chr3:34026676–34050970	XM_419554	similar to endoplasmic reticulum oxidoreductin 1- Lbeta
GgaAffx.22370.3.S1_s_at	4.3	0	0.27	chr3:1260579–1276286	XM_420116	similar to chromosome 17 open reading frame 28; downregulated in multiple cancer 1
GgaAffx.6334.2.S1_at	3.9	0.004	0.293	chr4:33725503–33732138	XM_420444	SH3 domain protein D19
GgaAffx.22790.1.S1_s_at	6.1	0.003	0.279	chr6:26875122–27047419	XM_421771	similar to actin-binding LIM protein 1 medium isoform
GgaAffx.26704.1.S1_at	-5.2	0.001	0.27	chr8:14622890–14638649	XM_422345	similar to abhydrolase domain containing 7
Gga.16560.1.S1_at	3.3	0.003	0.279	chrE26C13:1961–7813	XM_422853	myeloid cell leukemia sequence 1 (BCL2-related)
Gga.16020.1.S1_at	2.2	0	0.27	chrUn:92422001–92432256	NM_001031613	similar to Death-associated protein kinase 1

### Validation of gene expression data by quantitative real-time PCR

To validate the microarray results, we performed quantitative real-time PCR for: *propionyl-coenzyme A-carboxylase (PCC), similar to 1-phosphatidylinositol-4,5-bisphosphate phosphodiesterase gamma 2 (PBP2), tumor necrosis factor, alpha-induced protein 1 (TNFAIP1), fms-related tyrosine kinase 1 *(*FLT1), glycerol-3-phosphate dehydrogenase 2 (G3PD), low density lipoprotein-related protein 12 *(*LRP12), prostaglandin E receptor 3 (PER), suppression of tumorigenicity 7 (ST7), similar to endoplasmic reticulum oxidoreductin 1-Lbeta (ERO1), ataxin 3 *(*ATXN3), parvin, alpha (PARVA), CWF19-like 2 (CWF19), similar to Stxbp4 protein (Stxbp4), acyl-CoA oxidase (ACO)*, and *suppressor of cytokine signaling 7(SOCS7) *(Table 6). In all but one case (*TNFAIP1*), the real-time RT-PCR fold differences were in complete correspondence with the microarray data. Table 7 compares the microarray and real-time RT-PCR results.

**Table 6:** Primer pairs used to analyze gene expression by quantitative RT-PCR, and size of product

Gene	Forward primer^a^	Reverse primer^a^	Size
PCC	AGGGAAAGGTCGTGGCG	CCACTGCCAAGGACACTAGG	137 bp
PBP2	GAGTAAAGTCCGAGAACGAATG	AAATCCAGTAGTGGGACAAAGG	185 bp
TNFAIP1	TTCACTCTTAGAAATGCTGGTG	CTAAGTTAGTGGCAAAGCTGG	169 bp
FLT1	CTCTTTGGCATGAAAGGTGTC	CGTAGGTGTATCTTCGCTTGG	124 bp
G3PD	CTCCCATCCCATACCGACAG	GGCATATCGACTGCGTGTCC	167 bp
LRP12	GGAGTGTCAACGGCTTGTGG	ATCGGCGTGATCCCTGAACA	184 bp
PER	GGATCATGTGCGTCCTGTC	GGAGCAGCAGATAAACCCAC	210 bp
ST7	GTTCTATGTTGCCTTGACAGG	AAGTGGCTCACCGAGACCT	120 bp
ERO1	TTCAAGCCTCGATCTGTCTA	TCAAGAAGATAATTGGCACAC	182 bp
ATXN3	AAAGGTGACCTGCCAGAC	TTGCTTGGTCCACATCAC	142 bp
PARVA	GTGTACTTAGTCCTGCTAATGGG	TCTGGTCTGGGCTTTGGTT	161 bp
CWF19	ATCCCAGGGAAGTCTCGC	CTTTAGGCTCCTGTGGTTCAG	127 bp
Stxbp4	CCAAGATTTGAGAAAGAGGGTT	TCACTTAGAACAGCCGAGGAA	162 bp
ACO	GCTGTGCTCTATCAAGGTGGC	ACAAGGGCAACTGCGTCATC	110 bp
SOCS7	CATCCCAAGTTTGAAGACCG	CATTGCTGAACCTGGAGACG	163 bp
GAPDH	AGAACATCATCCCAGCGT	AGCCTTCACTACCCTCTTG	184 bp

a,: Sequences of oligonucleotides are indicated from 5' to 3' end

**Table 7:** Comparison of microarray and real-time RT-PCR analyses

Gene	Microarray^a^	Real-time RT-PCR	
	
	Fold change(F/L)^b^	Fold change(F/L)^b^	Student T-test^c ^(p-value)
PCC	2.5	2.45	0.0312*
ATXN3	2.7	4.47	0.0241*
PER	5.67	8.08	0.007**
ERO1	3.94	3.66	0.0308*
FLT1	4.21	4.46	0.0455*
CWF19	6.56	1.95	0.03*
PBP2	6.95	5.75	0.0521
PARVA	6.90	3	0.0221*
G3PD	7.46	2.32	0.0154*
Stxbp4	7.35	2.8	0.0031**
LRP12	1.82	8.83	0.0404*
ACO	2.19	2.76	0.2912
ST7	-2.1	-2.07	0.0042**
SOCS7	-10.0	-1.41	0.2117
TNFAIP1	-1.6	1.72	0.0086**

^a^: Microarray F/L fold changes are taken from Table 5.

^b^: Fold changes (F/L: mean of fat sample values on mean of lean sample values).

^c^:Student's t-test p-values, *p < 0.05, **p < 0.01.

## Discussion

Because *de novo *fatty acid synthesis in birds takes place mainly in the liver, most studies have been performed on hepatic tissues. The expression of some genes involved in lipid synthesis and secretion has been analyzed in the liver of lean and fat chickens. Bourneuf et al. [9] used a cDNA microarray to analyze the expression of genes in liver that are involved in pathways and mechanisms leading to adiposity, and found some genes differentially expressed between lean and fat chickens. Their research indicated that the mechanisms involved in the expression and regulation of lipogenic genes could play a key role in the ontogenesis of fatness in chickens from lean and fat lines. However, few studies have analyzed the functions of adipose tissue leading to adiposity in chickens.

Genome expression analysis aims to provide a broad and unbiased survey of the transcriptome, and requires true global coverage of a complex genome in a single microarray [10]. Significant progress has been made towards this goal and GeneChip microarrays have been improved significantly since they were invented [11].

The Chicken Genome Array, created by Affymetrix Inc. at the end of 2004 as part of the Ensembl annotation attempt at the complete chicken genome sequence (version 1, released May 2004), is a key research tool in chicken genomics. Our study provides, apparently for the first time, a comprehensive analysis of the chicken adipose tissue gene expression profile using this array. The gene expression profile results showed that 13,234–16,858 probe sets were detected in adipose tissue in 7-week-old chickens. Further study revealed that genes directly involved in lipid metabolism, including *Spot14, LPL, adrenomedullin, pyruvate dehydrogenase kinase, isoenzyme 4 *and *the FABP family*: *FABP 3 (muscle and heart), FABP 4 (adipocyte) and FABP 5 (psoriasis-associated)*, were highly expressed in adipose tissue; *FABP 4 *is the most highly expressed gene in this tissue. These genes are mainly involved in fatty acid transport and degradation. Other genes that are closely related to lipid metabolism and obesity by signal transduction or regulation of transcription were also highly expressed: *prosaposin (PSAP), retinol binding protein 7 (RBP7), retinoic acid receptor responder, IGFBP 7, thymosin beta 4, BMP7, superoxide dismutase 2 *and *annexin A5*. In addition, many genes involved in the immune response and cytophylaxis, such as *CD74 antigen, MHC class I glycoprotein, MHC class II beta 1 domain, MHC class II antigen alpha, lymphocyte antigen 6, beta-2 microglobulin, T-cell leukemia, Hsp70 *and *Hsp25*, were also highly expressed in adipose tissue. The most highly expressed genes (*A-FABP, LPL*) were apparently not differentially expressed between fat and lean lines. This may not preclude a major involvement of these factors in regulating lipid metabolism in chick adipose tissue, and further work on protein expression is needed to understand the metabolism of this tissue better. Our results demonstrated primarily that the functions of adipose tissue in chicken are important in lipid metabolism by directly or indirectly regulating the synthesis, transport and degradation of lipids. Moreover, adipose tissue may play an important role in chicken immunity and cytophylaxis. These results will be help to clarify the adipose tissue gene expression profile and to choose appropriate methods for further study of these genes.

On the other hand, genes described as "not expressed", including *obr*,*SREBP1*, *apoB*,*CCK*,*CCKR*,*IGF2 *and *lipoprotein (APOVLDLII)*, which have been studied extensively in other species (mainly *Homo sapiens *and *Mus*) and shown to affect obesity, were not detected in chicken adipose tissue in the present study. Possible reasons may be: (1) these genes are not expressed in chicken adipose tissue at all, or not at 7 weeks; (2) lipid metabolism in chicken is different from that in mammals. In birds, lipogenesis mainly occurs in the liver and is very limited in adipose tissue [12]. Although those genes were not detected in adipose tissue, they may regulate lipid metabolism indirectly. Another possible reason is gene interactions: the highly expressed genes may inhibit those with low expression levels and the "not expressed" ones when they participate the same biological responses.

Genetic variation in fatness was analyzed using two experimental lines (fat (FL) and lean (LL)) that were divergently selected for abdominal fat weight from a common genetic background. After selection for eight generations, the AFW and AFP differed significantly between the two lines (Figure 2). By consistency analysis within each line using hierarchical clustering of the total expressed genes, heterogeneity among individuals in each line was demonstrated (Figure 4). The hierarchical clustering results were barely satisfactory: the fat line showed higher consistency (about 87%) than the lean (about 77%). The consistency of each line is not sufficiently maintained. There may be two reasons for this. First, an insufficient number of generations have been used for selection, so the purity of each line will increase as selection continues. Second, our target trait is abdominal fat; after selection for eight generations the genes affecting abdominal fat may be better selected, but the genome array detects all genes expressed in adipose tissue, so genes affecting other traits, which have not been divergently selected, will reveal inter-individual differences within each chicken line. The higher consistency of the fat chicken line (about 87% compared to 77% for the lean line) shows that the fat line is purer than the lean line after being selected for eight generations. In addition, to make our study more reliable, the 7-3-16 individual was excluded from subsequent screening of differentially expressed genes because it deviated too much from the others.

Significance Analysis of Microarrays (SAM) has been considered a canonical algorithm for identifying differentially expressed genes in microarray data analysis. A total of 230 differentially expressed genes were screened by SAM. Fifteen genes that were differentially expressed between the two chicken lines were validated by real-time RT-PCR. To validate the results further, in addition to the samples used in the array experiments, four RNA pools (two from the fat line and two from the lean) were used for real-time RT-PCR analysis to reveal the differentially expressed genes more exactly. The results of this validation were encouraging: in all but one case (*TNFAIP1*), the real-time RT-PCR fold differences correlated well with the microarray data. Rajeevan et al. [13] evaluated the efficiency of real-time RT-PCR for validating the differentially expressed genes identified by microarrays. Their results indicated that genes showing less than a 2-fold difference in expression were not likely to be validated by real-time RT-PCR. Bourneuf et al. [9] considered that confirmation of genes with fold changes <1.7 by microarray analysis and/or <2 by real-time RT-PCR was difficult and likely to produce false positives. According to this report, the fold change of *TNFAIP1 *in the microarray was -1.6, which is difficult to confirm by real-time RT-PCR, so it is not correct to affirm that the two methods gave contradictory results for this gene.

Our analysis results revealed that the expression levels of some important genes implicated in lipid metabolism were up-regulated in fat chickens: *propionyl-coenzyme A-carboxylase, acyl-CoA oxidase, pyruvate dehydrogenase complex, glycerol-3-phosphate dehydrogenase, phosphatidylinositol, lysophospholipase 3, low density lipoprotein-related protein 12*, etc. Pyruvate dehydrogenase catalyzes oxidative decarboxylation of pyruvate to form acetyl-CoA, which is a central metabolite [14-16]. Acetyl-CoA enters the Krebs cycle, and is the donor of acetate for synthesis of fatty acids, ketone bodies and cholesterol. The expression level of *glycerol-3-phosphate dehydrogenase *increased in diet-induced obese animals [17]. *Propionyl-coenzyme A-carboxylase *is the key enzyme in the catabolism of odd-chain fatty acids, isoleucine, threonine, methionine and valine [18]. Phosphatidylinositol is an important lipid, both as a key membrane constituent and as a participant in essential signaling processes in all plants and animals. These genes regulate lipid metabolism mainly by enhancing the synthesis of fatty acids. In addition, the expression of genes involved in energy metabolism, such as *thioredoxin-like 4B, 5-oxoprolyl-peptidase*, etc., were markedly down-regulated in fat line chickens, and the expression of genes participating in gluconeogenesis or glycolysis, including *glycerol kinase (GK), ATPase, beta 1,3-galactosyltransferase *etc. were up-regulated in this line. In fact, it is well recognized that both energy metabolism [19] and glycometabolism [20,21] are highly related to obesity.

It is interesting that several genes in the protein tyrosine phosphatase pathway were more highly expressed in fat than in lean chickens: *fms-related tyrosine kinase 1, receptor tyrosine kinase flk-1/VEGFR-2, a type III receptor tyrosine kinase, protein tyrosine phosphatase-like, member b, protein-tyrosine phosphatase MEG2*, etc. This indicates that the protein tyrosine phosphatase pathway is very important in lipid metabolism, and further research is needed to elucidate the molecular mechanism.

Adipogenesis is promoted by the coordinated expression of many transcription factors [22,23]. De-differentiation, or loss of the adipocyte phenotype, has been observed in response to tumor necrosis factor and transforming growth factor β [24,25]. We also observed that several genes involved in tumorigenesis were differentially expressed between the two chicken lines. Expression of genes that inhibit tumorigenesis, such as *tumor necrosis factor, alpha-induced protein 1, suppression of tumorigenicity 7 *and *thrombospondin 1*, was down-regulated in fat line chickens, while the expression of genes promoting tumor formation, including *ret proto-oncogene *and *turban tumor syndrome*, was up-regulated. This indicates that tumorigenesis may be related to obesity. *TNFAIP1 *was first identified as a *tumor necrosis factor a (TNFa) *and *interleukin-6(IL-6) *inducible protein. It is induced rapidly and transiently by *TNFa *[26]. It is inferred that the *in vivo *effects of LPS on lipid metabolism are probably mediated by *TNFa*, which could be secreted by macrophages or by the adipose tissue itself [27,28]. Albalat et al. [29] suggested that *TNFa *could be a key modulator of lipid metabolism in fish. Taking these reports and our data into consideration, we presume that *TNFa *may play a significant role in chicken lipid metabolism.

## Conclusion

In conclusion, the chicken adipose tissue gene expression profile was investigated comprehensively in the present study, and specific genes that were differentially expressed in adipose tissue between a fat and a lean chicken line were identified with the aid of the Chicken Genome Array. Genes with high and low expression levels and "not-expressed" genes were identified, and 230 genes that were differentially expressed between the two chicken lines were screened out and confirmed by real-time RT-PCR. These genes were mainly involved in lipid metabolism, energy metabolism, signal transduction, tumorigenesis and immunity. Further analysis indicated that the pyruvate dehydrogenase complex, propionyl-coenzyme A-carboxylase, TNFa and the protein tyrosine phosphatase pathway may play key roles in lipid metabolism. If confirmed in future studies, these patterns of gene expression may contribute to understanding the molecular mechanism of obesity in chickens and provide potential targets for future therapy in humans.

## Methods

### Lean and fat chicken lines

The NEAU broiler lines divergently selected for abdominal fat content (NEAUHLF) have been selected since 1996 using percentage abdominal fat (%AFW) and plasma very low-density lipoprotein (VLDL) concentration as selection criteria. The G_0 _generation of the two lines came from the same grandsire line originating from the Arbor Acres breed, which was then divided into two lines according plasma VLDL concentration at 7 weeks. From G_1 _to G_8_, birds of each line were raised in two hatches. They were kept in similar environmental conditions and had access to feed and water *ad libitum*, and were fed with a commercial corn-soybean-based diet that met all NRC requirements (National Research Council, 1994). From hatching to 3 weeks the birds received a starter feed (3,100 kcal ME/kg and 210 g/kg CP), and from 4 weeks to slaughter they were fed a grower diet (3,000 kcal ME/kg and 190 g/kg CP). Plasma VLDL concentrations were measured for all birds at 7 weeks. Abdominal fat weight (AFW) of the male birds in the first hatch was measured and adjusted (%AFW) for body weight (BW) after slaughtering at 7 weeks. Birds with plasma VLDL concentration and AFP lower (lean line) or higher (fat line) than the population average value were selected as candidates for breeding, considering the body weights of male birds and egg production of female birds.

Selection was continued for 8 generations, with 15 sires and 4 hens per sire in the fat line and 10 sires and 4 hens per sire in the lean line for the G0 to G5 generation, then 25 sires and 4 dams per sire in each line for generations G6 through G8. The phenotypic correlation coefficient between body weight and AFW is 0.3789 (P < 0.01), and AFP is 0.1765(P < 0.01) at 7 wk of age based on data of eighth generation population, and the heritability of AFW and AFP were calculated based on the pedigree of eighth generation using the software MTDFREML. The heritability of AFW and AFP at G8 was considered high: AFW is 0.55, and AFP is 0.57.

### Sample preparation

Birds were slaughtered at 7 weeks and abdominal fat was isolated, immediately frozen in liquid nitrogen and stored at -80°C. The ten birds used in the present study were chosen by the percentage of abdominal fat (AFP): five had the highest AFP and the other five had the lowest (Table 1). Total RNA was extracted from 300 to 800 mg bulk abdominal adipose tissue using an RNeasy Lipid Tissue Mini Kit (QIAGEN, Valencia, CA) according to the manufacturer's recommendations, and quantified by spectrophotometry. mRNA was isolated using an Oligotex mRNA Mini Kit (QIAGEN, Valencia, CA). cDNA was prepared by oligo-dT-primed reverse transcription using Superscript II (Life Technologies, Inc.). Labeled cRNA probes were prepared using an IVT Labeling Kit (Affymetrix, Inc.) according to the manufacturer's protocol.

### Microarrays, hybridization and scanning

The GeneChip Chicken Genome Array used in present study was created by Affymetrix Inc. at the end of 2004, with comprehensive coverage of over 38,000 probe sets representing 32,773 transcripts corresponding to over 28,000 chicken genes. The Chicken Genome Array also contains 689 probe sets for detecting 684 transcripts from 17 avian viruses. Sequence information for this array was selected from the following public data sources: GenBank, UniGene (Build 18; May, 2004) and Ensembl (version 1, released on May, 2004).

Twenty micrograms of cRNA were fragmented at 94°C for 35 min in a 5 × fragmentation buffer containing 200 mM Tris-acetate (pH 8.1), 500 mM KOAc, 150 mM MgOAc. Prior to hybridization, the fragmented cRNA was heated at 95°C for 5 min in 1 × MES hybridization buffer (100 mM MES, 1 M NaCl, 20 mM EDTA, 0.01% Tween20) and 0.1 mg/ml herring sperm DNA, then at 45°C for 5 min, before loading on to the Affymetrix probe array cartridge (Affymetrix, Inc.). After prehybridization in 300 μl 1 × Hybridization Buffer at 45°C for 10 min, the Chicken Genome Arrays were incubated for 16 h at 45°C at constant rotation (60 rpm) using the manufacturer's hybridization buffer. Following hybridization, the arrays were washed with 6 × SSPE-T (0.9 M NaCl/60 mM NaH_2_PO_4_/6 mM EDTA/0.01% Tween20) at 25°C on a fluidics station (Affymetrix) for 10 × 2 cycles, then washed with 0.1 M MES/0.1 M NaCl/0.01% Tween20 at 50°C for 4 × 15 cycles. The arrays were stained with a streptavidin-phycoerythrin conjugate (Molecular Probes, Eugene, OR), followed by 10 × 4 wash cycles. To enhance the signals, the arrays were further stained with anti-streptavidin antibody for 10 min followed by a 10-minute staining with the streptavidin-phycoerythrin conjugate. After 15 × 4 additional wash cycles, the arrays were scanned at 560 nm using a confocal scanner (Affymetrix Gene array Scanner3000).

### Microarray data normalization

Raw data sets were normalized to total fluorescence, which represents the total amount of cRNA hybridized to a microarray, using the software Affymetrix Microarray Suite 4.0. Data sets were excluded if the absolute call (Abs Call) was A (absent) or M (Marginal) according to the detection *p*-value in all arrays. Only expressed transcripts (the Abs Call was P, present) were used in further analysis.

The raw data have been deposited in the National Center for Biotechnology Information (NCBI) Gene Expression Omnibus and have been assigned the following GEO accession numbers: GSM197816, GSM197817, GSM197818, GSM197819, GSM197820, GSM197821, GSM197822, GSM197823, GSE197824 and GSE197825

### Analysis of gene expression profile in 7-week broilers

The gene expression profile was investigated by three methods. First, according to the data normalization and Affymetrix Microarray Suite 4.0 software results, a gene was considered "not detected" by the scanner if its absolute call was A; we call these genes "not expressed" in this paper. The distribution of the expressed transcript (the Abs Call was P) was characterized by JMP4.0 according to its expression level, and genes with high and low expression levels were defined on the basis of this result.

### Hierarchical clustering analysis of the variation within each line

The fat and lean chicken lines have been selected for eight generations, so it is expected that the differences among individuals within each line are slight. Hierarchical clustering analysis of the genes expressed in five birds from each line was conducted to measure the consistency within each line. The value of R (coefficient correlation) was calculated and used to evaluate inter-line variation. Any individual that deviated too much from the others was excluded from the subsequent screening for differentially expressed genes.

### Identification of differentially expressed genes

Differentially expressed genes were identified from normalized data using the Significance Analysis of Microarrays (SAM) algorithm [8] implemented in a TIGR MultiExperiment Viewer. According to the SAM algorithm, genes are identified as differentially expressed on the basis of expression differences among the sample groups and the consistency of these differences; a score is assigned to each gene on the basis of a change in its expression relative to the standard deviation of repeated measurements for that gene. A gene is deemed 'significant' if its score surpasses a certain threshold. SAM calculates a false discovery rate (FDR), which is the median percentage of genes that are likely to be identified as significantly changed by chance. The threshold can be adjusted to identify different sets of putatively significant genes and the FDR is changed accordingly. The number of significantly changed genes in each experiment depends on a threshold with an acceptable FDR, selected by the investigator [8,30,31]. Clustering was achieved using uncentered Pearson correlations and average linkage clustering, and was displayed in TreeView. In the present study, a ?-value of 0.8 was chosen, giving a reasonable cutoff of 3.13 and 4.73 in d-scores.

### Real-time quantitative RT-PCR

Oligonucleotide primers were designed to amplify a fragment containing sequences from two adjacent exons in order to avoid contamination with genomic DNA. Glyceraldehyde-3-phosphate dehydrogenase (GAPDH) was considered to be a stably expressed housekeeping gene and was used as an internal reference gene. The primer pairs used to analyze gene expression and the size of product are shown in Table 6.

cDNA was prepared using an RNA PCR Kit (AMV) Ver3.0 (Takara) starting with 500 ng of total RNA from the following samples: (1) fat line: 7-3-3, 7-3-8, 7-3-15, 7-3-16, 7-3-24; (2) lean line: 7-1-1, 7-1-2, 7-1-4, 7-1-11, 7-1-17; (3) four RNA pools (two from each line), each containing three samples that were not used for the array experiments. First-strand cDNA synthesis was performed at 42°C for 30 min. The 10 μl reaction mixture also contained 1 × AMV reaction buffer, 1 mM each dNTP, 0.125 pmol oligo-dT-adaptor primer, 5 mM MgCl2, 10 U RNase inhibitor, and 2.5 U avian myeloblastosis virus (AMV) reverse transcriptase XL. After incubation, the mixture was heated at 99°C for 5 min to extinguish reverse transcriptase activity. Relative quantification of the expression of selected genes was performed using SYBR^® ^Premix Ex Taq™ (Takara). The reaction mixtures were incubated in an ABI Prism 7300 Sequence Detection System (Applied Biosystems) programmed to conduct one cycle at 95°C for 10 s and 40 cycles at 95°C for 5 s and 60°C for 31 s. The dissociation curves were analyzed using Dissociation Curve 1.0 software (ABI) for each PCR reaction to detect and eliminate possible primer-dimer artifacts. Results (fold changes) were expressed as 2^ΔΔCt ^with ΔΔ*Ct *= (*Ctij *- *CtGAPDHj*) - (*Cti1 *- *CtGAPDH1*), where *Ctij *and *CtGAPDHj *are the *Ct *for gene i and for GAPDH in a pool or a sample (named j), and *Cti1 *and *CtGAPDH1 *are the *Ct *in pool 1 or sample 1, expressed as the standard [9].

## Abbreviations

ACO: acyl-CoA oxidase; ACX3: acyl-CoA oxidase; AFW: abdominal fat weight; AFP: percentage of abdominal fat; ApoB: apolipoprotein B; ATXN3: ataxin 3; BW: body weight; CCK: cholecystekinin; CCKR: cholecystekinin receptor; CWF19L2: CWF19-like 2, cell cycle control (S. pombe); ERO1: similar to endoplasmic reticulum oxidoreductin 1-Lbeta; FABP: fatty acid binding protein; FLT1: fms-related tyrosine kinase 1; G3PD: glycerol-3-phosphate dehydrogenase 2; IGF2: insulin-like growth factor 2; IGFBP7: insulin-like growth factor binding protein 7; LPL: lipoprotein lipase; LRP12: low density lipoprotein-related protein 12; NEAU: Northeast Agricultural University; PARVA: parvin, alpha; PBP2: similar to 1-phosphatidylinositol-4,5-bisphosphate phosphodiesterase gamma 2; PCC: propionyl-coenzyme A-carboxylase; PER: prostaglandin E receptor 3; SAM : Significance Analysis of Microarrays; SOCS7: Suppressor of cytokine signaling 7; Spot14: thyroid hormone-responsive protein; SREBP: sterol regulatory element binding proteins; ST7: suppression of tumorigenicity 7; Stxbp4: similar to Stxbp4 protein; TNFAIP1: Tumor necrosis factor, alpha-induced protein 1.

## Authors' contributions

HBW contributed to the design of the microarray, participated in the interpretation of data, performed the verification of microarray data, drafted and wrote the manuscript. HL co-led the conception and design of the study, contributed to the design of the microarray, participated in the interpretation of data, and contributed to writing the manuscript. QGW participated in the design of the study and interpretation of data, and contributed to writing the manuscript. XYZ carried out analysis and interpretation of microarray data and assisted in production of the microarray. SZW participated in the design of the study and contributed to writing the manuscript. YXW participated in the design of the study and contributed to writing the manuscript. XPW performed microarray hybridizations, generated microarray data, and assisted in production of the microarray. All authors submitted comments on drafts, and read and approved the final manuscript.
